# Dragonfly wing decorated by gold nanoislands as flexible and stable substrates for surface-enhanced Raman scattering (SERS)

**DOI:** 10.1038/s41598-018-25228-8

**Published:** 2018-05-02

**Authors:** Guo Chao Shi, Ming Li Wang, Yan Ying Zhu, Lin Shen, Wan Li Ma, Yu Hong Wang, Rui Feng Li

**Affiliations:** 1 Key Laboratory for Microstructural Material Physics of Hebei Province, School of Science, Yanshan University. Qinhuangdao, Hebei, 066004 PR China; 2 College of Liren, Yanshan University, Qinhuangdao, Hebei, 066004 PR China; 3 Department of Mathematics, NC State University, Raleigh, 276968205 USA

## Abstract

A flexible and stable biomimetic SERS substrate was successfully fabricated by depositing gold (Au) nanoislands on the dragonfly wings (DW) via a simple DC magnetron sputtering system. Characterizations of the Au/DW nanostructure indicated that the optimum Au/DW-45 (sputtering time was 45 min) substrate owns high sensitivity, good stability and outstanding reproducibility. The limit of detection (LOD) for Rhodamine 6 G (R6G) was as low as 10^−7^ M and enhancement factor (EF) was calculated to be 2.8 × 10^6^. 70-day-duration stability tests showed that Raman intensity of R6G reduced only by 12.9% after aging for 70 days. The maximum relative standard deviations (RSD) of SERS intensities from 100 positions of Au/DW-45 substrate were less than 8.3%, revealing outstanding uniformity and reproducibility. Moreover, the flexible Au/DW-45 bioscaffold arrays were employed to solve the vital problem of pesticide residues. By directly sampling from tomato peels via a “press and peel off” approach, cypermethrin has been rapidly and reliably determined with a LOD centered at 10^−3^ ng/cm^2^ and a correlation coefficient (R^2^) of 0.987. The positive results demonstrated that the Au-based DW biomimetic arrays may offer an efficient SERS platform for the identification of various pesticide residues on real samples.

## Introduction

Recently, high-performance detection technique of surface-enhanced Raman scattering (SERS) has been widely applied in chemical and biological sensing due to its high sensitivity, rapid response and nondestructive testing process^1–3^. Because the SERS technique enables spectrum analysis by detecting the vibrational bands of the adsorbed molecules, this technique can provide fingerprint information for numerous molecules and even achieve single molecule detection^4,5^. It has been widely acknowledged that the large enhancement effect of SERS technique is primarily due to the electromagnetic enhancement (EM) mechanism and chemical enhancement (CM) mechanism. The EM mechanism, which results from the amplification of light by the excitation of localized surface plasmon resonance (LSPR) at appropriate nanogaps (“hot spots”) between metallic nanostructures is commonly accepted to be the origin of SERS^6,7^. Au, silver (Ag) and copper (Cu) nanostructures which reveal high performances in EM are the most popular SERS platforms according to the previous studies^8–12^. As regard to the SERS enhancement performance, Ag is most widely used by researchers in SERS detection for its large optical response in the visible range, efficiency in enhancing the Raman scattering and simple structure for geometric modulation. However, the main drawback of Ag substrate is that it is easy to suffer from oxidation which limits its application in SERS field. Cu is the cheapest among the three metallic materials, however, its SERS enhanced effect is the weakest. Au, which has more stable chemical properties than Ag, announces an outstanding application in organic pollutant detection^13^. Furthermore, Au also has a good biological compatibility as well as a strong and tunable LSPR in visible and near-infrared spectral regions and therefore is the ideal candidate material for our substrate^14^. It has been widely confirmed that the morphology and structure of the enhancing surface plays the most important role in the high-performance detection. The nanostructures of the Au-based substrates included nanowires^15^, nanopillars^16^, nanorods^17^, nanostars^18^ and core-shell structure^19^ have been reported. These 3D nanostructures with abundant nanogaps not only provide more tremendous EM enhancement in the gaps between Au nanostructures than those of one-dimensional^20^ and two-dimensional^21^ nanostructures, but also increase appropriate sites for probing molecules within the laser footprint. Although many synthesis technologies such as chemical reduction impregnation method, seed-growth synthesis, electron beam lithography, self-assembly and indentation lithography have been achieved using Au nanostructures as SERS substrates, the major shortcomings of these methods are also noticeable. Generally, these approaches usually require complicated preparation processes, stringent laboratory conditions as well as high fabrication cost, which limit the further development of the SERS technique. Therefore, seeking an economical and manageable method to prepare SERS-active substrates has attracted more and more attention.

Recently, an increasing number of researchers have paid extensive attention to combining noble metal materials with super-hydrophobic biomaterials (e.g. plant leaves, insect wings) to fabricate SERS substrates. Surprisingly, these rough biomaterials show a high-performance enhancement as SERS substrates after decorated with noble metal nanoparticles. Besides, the surfaces of the substrates are still super-hydrophobic, ensuring a large number of probe molecules to locate in a tiny area after natural evaporation^22^. For example, based on physical vapor deposition (PVD) method, silver nanoparticles with diameter of 20 to 30 nm were successfully coated on the surface of the natural rose petal and its LOD for R6G was 10^−9^ M^23^. Kumar *et al*.^24^ proposed a highly sensitive and flexible SERS sensor based on Ag decorated on the surface of taro leaf. A SERS enhancement factor of 2.06 × 10^5^ was obtained and the LOD for the malachite green was 10^−11^ M. Mu and his co-workers developed a low-cost and green method to fabricate SERS substrates by *in situ* reducing gold nanoparticles in different butterfly wings and gained satisfactory results^25^. Hence, dragonfly wings (DW) with super hydrophobic surface and irregular nanopillars microstructures were employed by our group to prepare sensitive and stable SERS-active substrates.

In this paper, we demonstrated a manageable fabrication process of Au nanoislands in different sizes on the surface of DW arrays via DC magnetron sputtering technique. Compared with other types of SERS substrates, the as-prepared Au/DW substrate was flexible, sensitive, low-cost and stable. The influence of Au sputtering time was investigated and analyzed. Our analysis suggested that the Au/DW substrate with the sputtering time of 45 min achieved the best enhancement and the LOD for R6G was as low as 10^−7^ M. Meanwhile, the enhancement mechanism of Au/DW-45 substrate was simulated by the method of 3D finite-difference time-domain simulation based on its microstructure characteristics. For the application of Au/DW-45 substrate, it was used to detect a kind of insecticide called cypermethrin via a simple “press and peel off” method from tomato peels and the LOD was located at 10^−3^ ng/cm^2^. This work indicated that Au/DW-45 substrate has potential in rapid sampling and analyzing multiple pesticide residues in various food products.

## Experimental

### Materials and Instruments

The sputtering target of gold (99.99%) was obtained from ZhongNuo Advanced Material (Beijing) Technology Co., Ltd. DWs were supplied by Hebei University of Environmental Engineering. Experiments with dragonfly wings complied with the accepted ethical stsndards and were approved by the Ethical Review Board of Yanshan University on 15 June 2017. Acetone, ethanol were supplied by Key Laboratory for Microstructural Material Physics of Hebei Province. R6G and cypermethrin were analytical grade and obtained from J&K Scientific LTD. Other reagents used in the experiments, unless mentioned otherwise, were of analytical grade and used without further purification. Deionized water (15.6 MΩ) was used for all solution preparations.

The dragonfly wings were coated by Au nanoislands in the high vacuum DC magnetron sputtering system (LAB 18) and the deposition rate was 0.035 nm/s to fabricate Au nanoislands with different sizes. The surface morphology and size distribution of the Au nanoislands on different substrates were characterized by field emission scanning electron microscopy (FE-SEM) (JEOL JSM-2100). Raman spectra of R6G and cypermethrin were obtained by Raman system (inVia). During the Raman detection, the numerical aperture was 0.75 and the objective was ×50. The UV-vis absorption spectra were monitored by Shimadzu UV-3600 UV-vis spectrophotometer.

### Sample preparation

**Figure 1:**
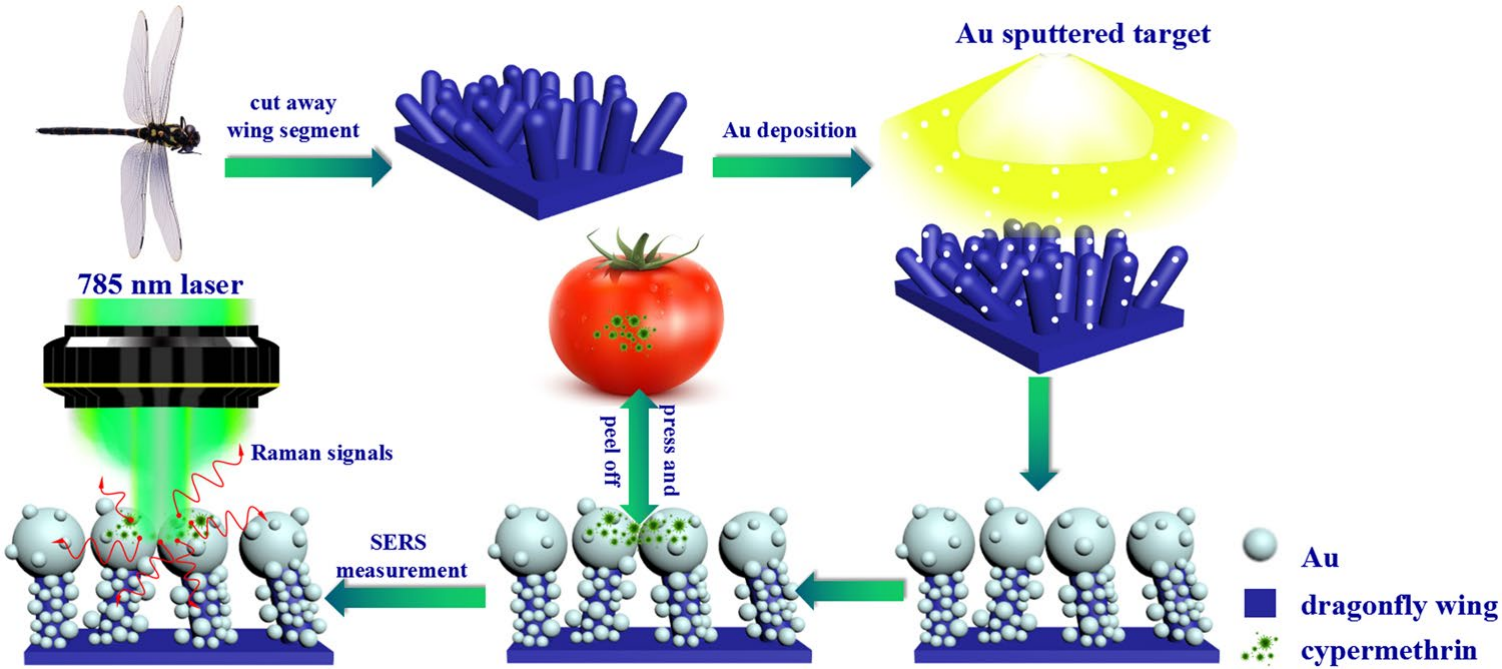
Schematic illustration of the fabrication process of the SERS substrates through sputtering Au on dragonfly wing by DC magnetron sputtering system and SERS measurement of Au/DW-45 substrate by Raman system.

Before the fabrication of Au/DW substrates, a series of dragonfly wings with an area of 1 cm × 1 cm were cleaned by acetone, ethanol and deionized water for 20 min in turn to remove the residual impurities, followed by natural drying. The DW we used in the experiment is called *pantala flavescens* which belong to *Anisoptera, Libellulidae*^26^. For a straightforward comparison, the cleaned DWs were adhered to the blank silicon wafers which made the SERS substrates easier to handle. Afterwards, the Au nanoislands were deposited on the DW surface by the high vacuum DC magnetron sputtering system with 10^−5^ mbar base pressure as shown in Fig. 1. The power supply was 70 W which was operated at a crystal-controlled frequency of 13.56 MHz. After pumping the sputtering Argon gas with 99.9% high purity, the vacuum degree of sputtering chamber was controlled at 4.5 × 10^−3^ mbar. The circular sputtering target gold (99.99%) with a diameter of 50.8 mm was used in the experiments. The thickness of the target was 3.175 mm. During the sputtering process, the deposition time of Au nanoislands was controlled to be 15 min, 30 min, 45 min and 60 min and the sputtering rate was set to be 0.035 nm/s calibrated by a spectroscopic ellipsometer. Consistently, these SERS substrates were signed with Au/DW-15, Au/DW-30, Au/DW-45 and Au/DW-60 in the following discussion. All the sputtering deposition processes were performed at room temperature.

### SERS measurements

A Raman system (inVia) was used to record the Raman spectra. As shown in Fig. 1, the line laser with the wavelength of 785 nm was chosen as an excitation light source which generated the weakest fluorescent signals compared to 532 nm and 633 nm laser. The excitation beam with a spot size of ca 1 μm was focused onto the samples and the spectral resolution was 1 cm^−1^. The incident laser power was kept at 0.5 mW to avoid any damage of the samples. Due to the high Raman scattering cross-section for SERS measurement, R6G fluorescent dye was used as the probe molecules. Prior to the measurements, a droplet of 10 μL R6G with different concentrations (10^−1^ M–10^−7^ M) was deposited onto the as-prepared Au/DW substrates. The samples with R6G droplet were dried in a vacuum drying oven at 40 °C for 5 min. The exposure time for each SERS measurement was typically set to be 10 s. Unless otherwise specified, the accumulation time and the laser power were the same for all Raman spectra. The condition of the cypermethrin SERS measurement is similar to the R6G detection.

### Collection of cypermethrin on tomato peels

In this report, tomatoes were cleaned three times with deionized water and ethanol before used to eliminate the effects of pollutants on the results of the experiment. Then, cut the cleaned tomatoes into nearly uniform squares of 1 × 1 cm^2^. Afterward, 10 μL of the prepared cypermethrin solutions with different concentrations (10 ng/cm^2^–10^−3^ ng/cm^2^) were directly sprayed onto the surface of the tomato peels. After drying at room temperature, 10 μL of ethanol solution was spread on the area sprayed with cypermethrin solutions. Finally, the SERS substrate was pressed to the samples until it was dried followed by peeling off for further SERS analysis as shown in Fig. 1. To insure that the Raman signals of cypermethrin molecules were successfully collected, the SERS measurements were performed 2 times randomly within the treated area and the collected SERS data were averaged.

## Results and Discussion

### Characterization

**Figure 2:**
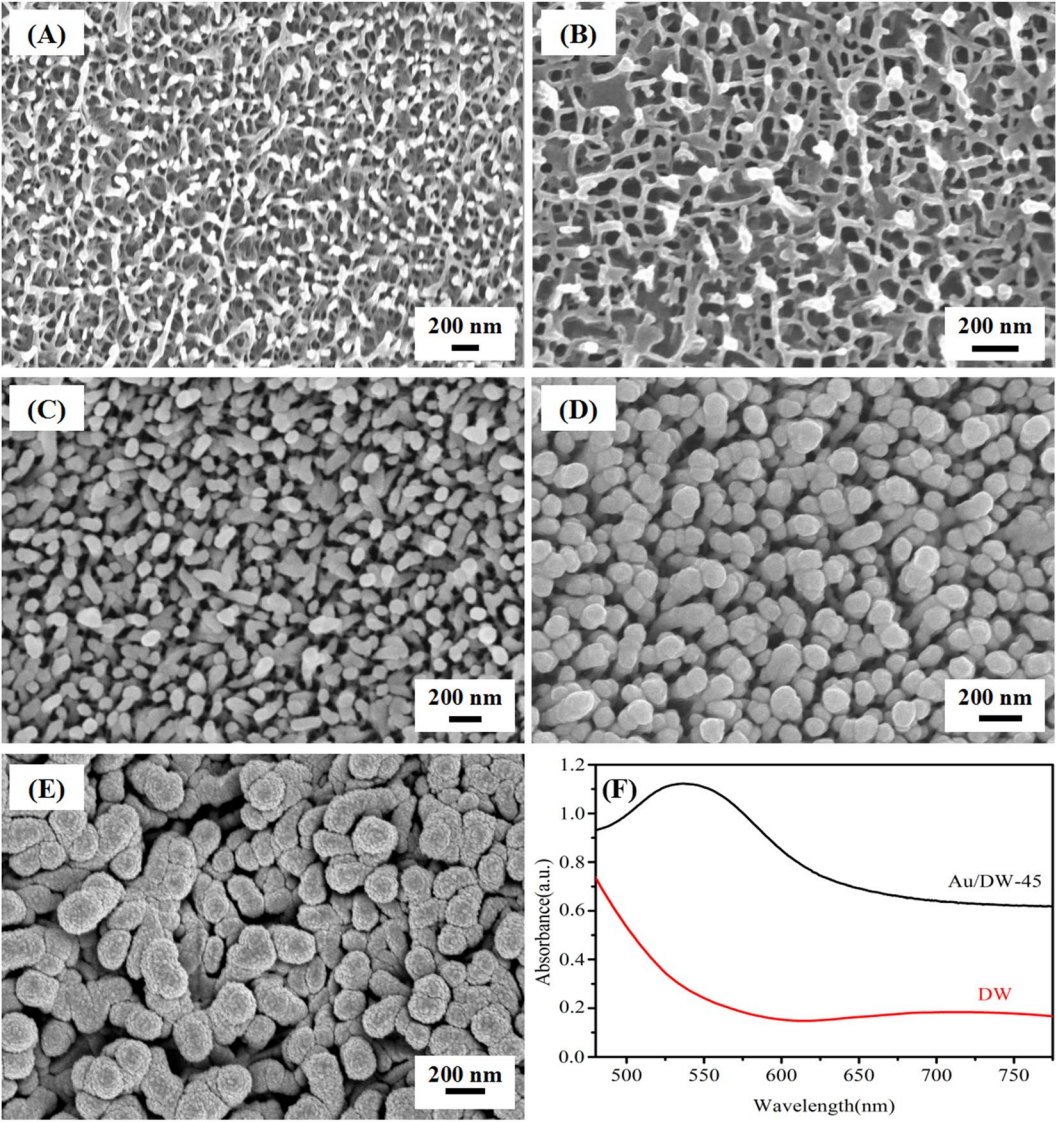
(**A**) FE-SEM images of DW from the top-view; FE-SEM images of Au/DW substrates obtained from different sputtering time: (**B**) Au/DW-15 substrate, (**C**) Au/DW-30 substrate, (**D**) Au/DW-45 substrate and (**E**) Au/DW-60 substrate, respectively; (**F**) UV-vis spectra of DW and Au/DW-45.

As we can see from the FE-SEM image in Fig. 2(A), a large number of multi-column nanopillars existed on the epicuticular layers of the dragonfly wings and they were randomly distributed. The nearest-neighbor nanopillars distance was approximately 180 ± 30 nm, the average height of the nanopillars was about 200 nm and the average diameter of the round tops was approximately 80 ± 20 nm, respectively^27^. Fig. 2(B–E) shows the FE-SEM images of Au/DW substrates obtained from different sputtering time. It was easily observed that the sputtering time could greatly affect the morphologies. The FE-SEM image of as-deposited Au/DW-15 substrate was shown in Fig. 2(B). From this, we can observe that a number of Au nanoislands with an average diameter of 31.5 ± 5 nm which calculated by the sputtering time (15 min) and the rate (0.035 nm/s) were successfully decorated on the tops of nanopillars without damaging the original morphology. Figure 2(C) presents the FE-SEM image of Au/DW-30 substrate, we can also observe that the Au nanoislands were deposited on the surface of DW with the average size of 63 ± 5 nm. Obviously, the deposited Au nanoislands were grown or distributed uniformly according to the shape of the nanopillars. As shown in Fig. 2(D), when the sputtering time increased to 45 min, the nanopillar-like structures disappeared and the nanorough Au nanoislands with the average diameter of 95 ± 2.5 nm were formed on the tops. Meanwhile, the side of the nanopillars were covered with Au films. This image was a representative one taken at different regions of the Au/DW-45 substrates. Clearly, the distribution of the Au nanoislands is more homogeneous on the tops of the nanopillars and there were many suitable nanogaps between the Au nanoislands for the enrichment of the probe molecules, which will further enhance the scattering cross-section and contribute to the homogeneity of the SERS signal. As the sputtering time increased to 60 min, the anisotropic growth of nanoislands was suppressed and the formation of Au nanoislands on the spheroidal shape. As shown in Fig. 2(E), the surface of the DW was almost fully covered by the spheroidal nanoislands with the average size of 126 ± 10 nm which resulting in a sharp decline of SERS enhancement. As shown in Fig. 2(F), the Au/DW-45 substrate exhibited obvious enhanced capability of light absorption in the range of 450–775 nm in comparison to the untreated DW. Moreover, after the sputtering of Au nanoislands, a broad absorption covering the range of 500–650 nm with a summit at around 550 nm appeared, indicating the formation of Au nanoislands onto the DW^28^. Although the wavelengh of exciting source (785 nm) is not matched with surface plasmon band (550 nm), the as-prepared Au/DW-45 SERS substrate showed strong Raman signals, which may attribute to the rough indication on matching between exciting source and surface plasmon resonance of UV-vis absorption spectra^29,30^.

### SERS performances and EF calculation

**Figure 3:**
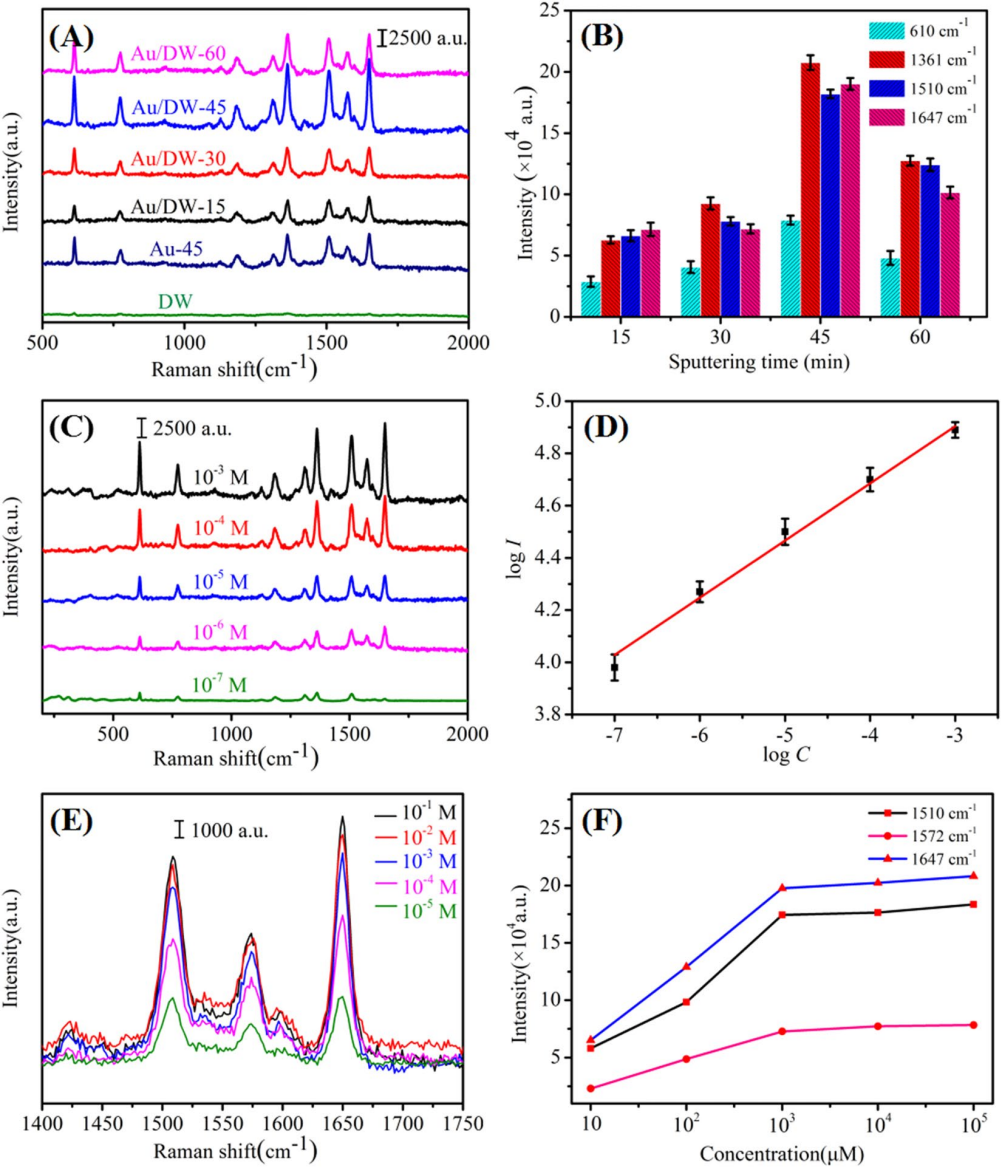
(**A**) Raman spectra of 10^−3^ M R6G adsorbed on Au/DW substrates prepared with different sputtering time (15 min, 30 min, 45 min and 60 min); (**B**) Raman intensity at 610, 1361, 1510 and 1647 cm^−1^ as a function of sputtering time (the error bars were calculated based on 10 independent measurements); (**C**) SERS spectra of R6G with different concentrations from 10^−3^ M to 10^−7^ M on Au/DW-45 substrate; (**D**) linear calibration plot between the SERS intensity and R6G concentration in the logarithm scale (the error bars were calculated based on 10 independent measurements); (**E**) SERS spectra of R6G collected from the Au/DW-45 substrate with different concentrations from 10^−5^ to 10^−1^ M; (**F**) integrated peak intensity (*I*_*SERS*_) of 1510, 1572 and 1647 cm^−1^ Raman bands corresponding to (**E**).

As previously reported, the tremendous enhancement of the Raman signal only occurred when the probe molecules were directly (or closely) adsorbed on the surface of the noble metal^31^. So the strong affinity between the probe molecules and the active substrate is critical to achieve high SERS sensitivity. Due to the well-established vibrational features of R6G, SERS performances of Au/DW substrates were functionalized by R6G^32^. Fig. 3(A) shows the SERS spectra of 10^−3^ M R6G adsorbed on different substrates prepared by different sputtering time. Raman signals observed at about 610, 774, 1187, 1310, 1361, 1510, 1572 and 1647 cm^−1^ were assigned to the characteristic vibrational frequencies of R6G^33^. The prominent peak at 610 cm^−1^ was attributed to C-C-C ring in-plane bending mode, the peak at 774 cm^−1^ was related to C-H out-of-plane bending mode and 1187 cm^−1^ was associated with C-C stretching vibrations mode, respectively. Other fingerprint peaks at about 1310, 1361, 1510, 1572 and 1647 cm^−1^ corresponded to symmetric modes of C-C stretching in-plane vibrations of R6G because of the LSPR effect excited by the 785 nm laser^34^. In addition, the intensities of the Raman signals were different among these four types of Au/DW substrates. Raman intensities of R6G on Au/DW-15 substrate and Au/DW-30 substrate were weak. Surprisingly, when the sputtering time increased to 45 min, the Au/DW-45 substrate exhibited much stronger SERS intensity than those obtained from Au/DW-15, Au/DW-30 and Au/DW-60 substrates. Meanwhile, referenced spectra of 10^−3^ M R6G obtained from Au-45 substrate which prepared by directly sputtering Au onto a Si wafer and from neat DW substrate were also plotted in Fig. 3(A). It is indicated that DW played an important role in SERS enhancement. In order to analyze quantitatively the effect of sputtering time on the SERS performance, Raman intensities at 610, 1361, 1510 and 1647 cm^−1^ as a function of the sputtering time were shown in Fig. 3(B). Choosing the wavenumber of 1361 cm^−1^ for example, as the sputtering time increased from 15 min to 60 min, the Raman intensities got intense firstly and then faded down. Raman intensity from Au/DW-45 substrate was 3.3, 2.2 and 1.62 times than that from Au/DW-15, Au/DW-30 and Au/DW-60 substrates, respectively. All these results demonstrated that proper sputtering time increases the Raman intensity, resulting in a large enhancement of the SERS performance of Au/DW substrates.

One of the major aims of this study is to develop an Au/DW substrate for sensitive SERS detection. Here, the Au/DW-45 hybrid as optimized SERS substrate was chosen to test its sensitivity response to R6G with the concentrations from 10^−3^ M to 10^−7^ M. As shown in Fig. 3(C), the measured Raman intensities decayed with the decreased of the R6G concentrations. The characteristic peaks at 610, 774, 1310, 1361, 1510 and 1572 cm^−1^ could still be identified even at a low concentration of 10^−7^ M which indicated that the LOD for R6G was estimated to be about 10^−7^ M. This lower LOD was probable on account of the super-hydrophobicity of DW. When a drop of 10 μL R6G solution was deposited on the Au/DW-45 substrate which also possessed of super-hydrophobic surface, the droplet formed to be a round-likely roundness^30^. After natural evaporation, the probe molecules in solution would be inspissated into a certain region, which further increased the LOD of the Au/DW-45 substrate^35^. To demonstrate the quantitative detection capability of Au/DW-45 substrate, the Raman intensities centered at 610 cm^−1^ as a function of R6G concentrations were depicted in Fig. 3(D). The average intensities was based on ten spectra randomly collected on Au/DW-45 substrate. When the concentrations and the intensities were transformed into a logarithm scale, the response between log *C* and log *I* was nearly linear and the value of R^2^ was as high as 0.991. This reasonable linear results further prove that this strategy based on Au/DW-45 substrate prepared by DC magnetron sputtering system has a strong application potential for the rapid detection of unknown concentrations of R6G solution.

Calculating the enhancement factor (EF) value was a typical approach to evaluate the SERS performance of a substrate. The EF for the 3D Au/DW-45 substrate was calculated using the following formula^36^:

1 $${\rm{EF}}=({I}_{SERS}/{I}_{bulk})\times ({N}_{bulk}/{N}_{SERS})$$

where the *I*_*SERS*_ is the integrated SERS intensity for the probe molecules of 10^−3^ M R6G on the surface of Au/DW-45 substrate, *I*_*bulk*_ is the measured intensity of 10^−3^ M R6G on a DW substrate, *N*_*bulk*_ and *N*_*SERS*_ are the number of the R6G molecules absorbed on the DW substrate and Au/DW-45 substrate under the laser spot area, respectively. Here, the peak of R6G at 1647 cm^−1^ was chosen for *I* value calculation. When a droplet of 10^−3^ M R6G absorbed on the Au/DW-45 substrate and DW substrate, the *I*_*SERS*_ and *I*_*bulk*_ were measured to be 2.08 × 10^5^ and 268. Therefore, the ratio of *I*_*SERS*_*/I*_*Raman*_ was calculated to be 776.1. *N*_*bulk*_ was calculated according to the standard formula^37^:

2 $${N}_{bulk}=A\times h\times {c}_{bulk}\times {N}_{A}$$

where *A* is the area of the laser focal spot, *h* is the confocal depth of the laser, *c*_*bulk*_ is the concentration of R6G bulk solution and *N*_*A*_ is the Avogadro constant, respectively. In the test, the laser focal spot had a diameter of 1 μm, the confocal depth of the 785 nm laser was about 3 mm. Therefore, the estimated value of *N*_*bulk*_ could be 1.42 × 10^9^. We measured *N*_*SERS*_ on the assumption that R6G molecules were in monolayer adsorption on the Au/DW-45 SERS substrate. According to previously reported, the surface area of one R6G molecule was calculated by its length (1.37 nm) multiplied by its width (1.43 nm)^10^. Figure 3(E) shows the Raman spectra of R6G obtained from the Au/DW-45 substrate with different concentrations from 10^−5^ M to 10^−1^ M, and Fig. 3(F) presents the corresponding peak intensities (*I*_*SERS*_) of the 1510, 1572 and 1647 cm^−1^ Raman bands. Obviously, the values of the *I*_*SERS*_ enhanced with the increase of the R6G concentrations from 10^−5^ M to 10^−3^ M. However, the *I*_*SERS*_ of 10^−2^ M and 10^−1^ M were very close to that of 10^−3^ M. This phenomenon indicated that the surface of the Au/DW-45 substrate was presumed to be fully adsorbed with R6G molecules when the concentration reached 10^−3^ M^17^. Subsequently, dividing the laser facula area by the surface area of a single R6G molecule, the *N*_*SERS*_ was calculated to be 3.93 × 10^5^. According to the formula (1), the 3D Au/DW-45 substrate possesses a large EF of approximately 2.8 × 10^6^. The EFs calculated from other Raman bands were shown in Table 1, revealing high SERS enhancement of Au/DW-45 substrate.

**Table 1:** Calculated EFs based on the Au/DW-45 substrate and DW substrate of 10^−3^ M R6G absorbed.

Peak(cm^−1^)	611	774	1187	1361	1510
EF	1.05 × 10^6^	1.24 × 10^6^	1.86 × 10^6^	1.20 × 10^6^	1.85 × 10^6^

The calculated high EF of the Au/DW-45 substrate may be ascribed as follows. Firstly, from the perspective of structural analysis of 3D Au/DW-45 substrate, the irregular nanopillars on the surface of the DW owned huge specific surface area, which offered a larger surface area for the sputtering of Au nanoislands and for much more adsorption of R6G molecules. Secondly, the 3D array of Au/DW-45 substrate had a good “light trap” effect. When laser entered the arrays, both the incident and scattered light enhanced, which further contributed to the EM enhancement at the interfaces^38^. Thirdly, the Au nanoislinds decorated on the titled nanopillars was made up of close-packed Au nanoparticles with close nanogaps, where high-density “hot spots” were formed. When excited by the incident light, the LSPR effect was significantly enhanced in a small volume of several cubic nanometers in the surface regions^39^. Therefore, it is not surprising to observe that Au/DW-45 substrate exhibits high Raman intensity enhancement.

### 3D finite-difference time-domain simulation

**Figure 4:**
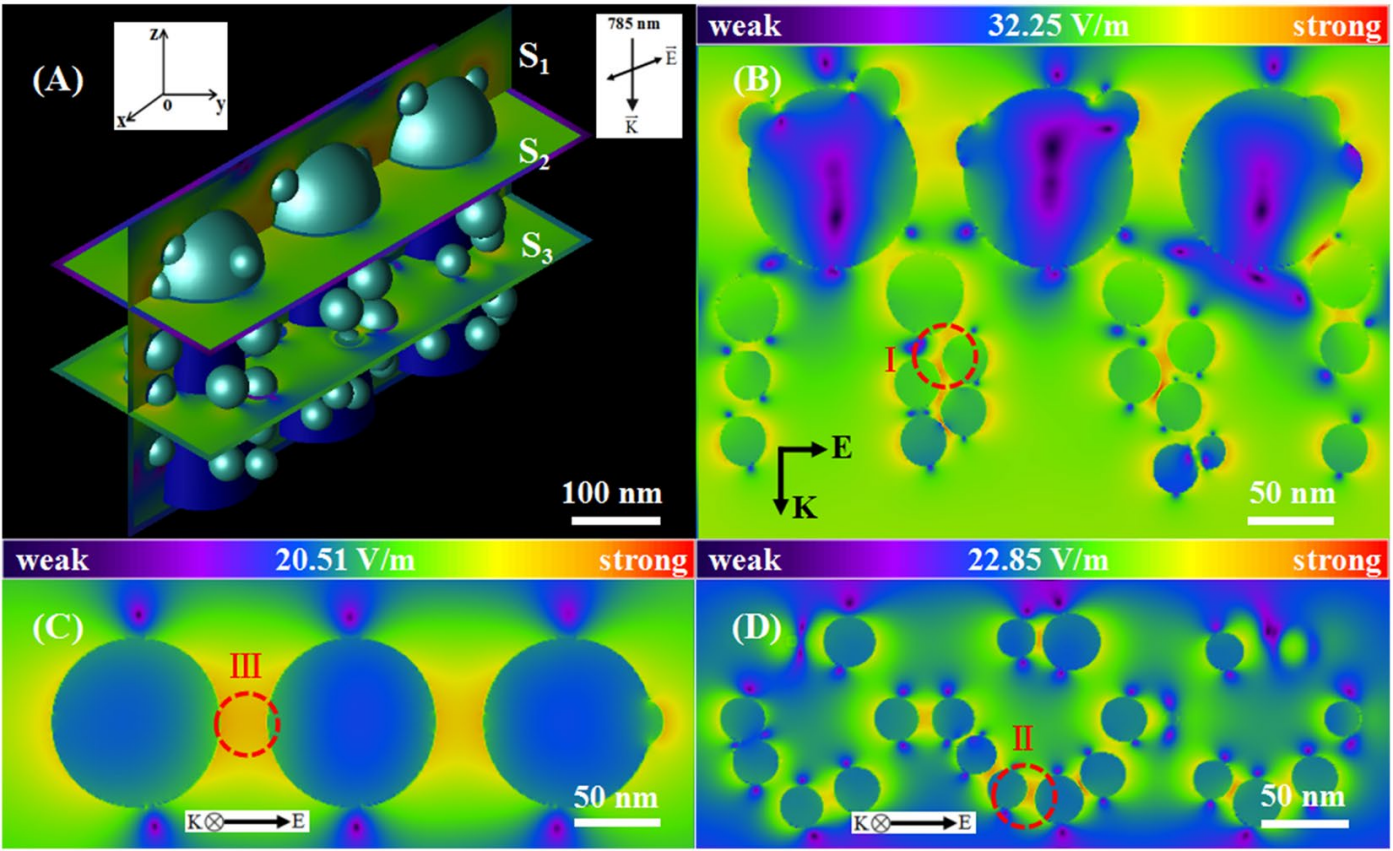
(**A**) Shape of the 3D-FDTD model of Au/DW-45 substrate; (**B**), (**C**) and (**D**) the calculated spatial electric field distribution intensity for the planes S_1_, S_2_ and S_3_ in (**A**).

In order to further explore and understand the excellent SERS behaviors of the Au/DW-45 substrate, we simulated and analyzed the spatial distribution of the local electric fields by employing the 3D finite-difference time-domain (3D-FDTD) simulation method. Based on the morphological structure of the Au/DW-45 substrate, the structural model is presented in Fig. 4(A), where 95 nm Au nanoislands were decorated on the top of nanopillars and other varisized ones were decorated on the side surfaces. As stated above, the height, the top diameter and the top spacing of the nanopillars were 200 nm, 80 nm and 180 nm, respectively. In addition, a square-shaped continuous wave laser with a wavelength of 785 nm was elected as the incident light in our work. The direction of the laser was propagated along the *z* direction and the direction of polarization was perpendicular to *z* direction. Fig. 4(B–D) present the distributions of electrical field intensity of the planes of S_1_, S_2_ and S_3_ defined in Fig. 4(A). Obviously, a large number of “hot spots” resided in 3D Au/DW-45 substrate under the 785 nm excitation, where these “hot spots” corresponded to the locations of the three types of nanogaps. Type “I” was formed between the Au nanoislands on the two neighboring nanopillars. Type “II” presented between the Au nanoislands on the same nanopillars. Type “III” existed in the nearest-neighbor Au nanoislands on the top of the neighbouring nanopillars. The maximum value of the local electric field intensity for the model of Au/DW-45 substrate was 32.25 V m^−1^. To directly compare the EFs obtained from the 3D-FDTD simulations and calculated from the SERS experiments, the simulative EF was calculated according to the following formula (3)^40^:

3 $${{\rm{G}}}_{{\rm{SERS}}}={|{E}_{loc}(\omega )/{E}_{inc}(\omega )|}^{{\rm{4}}}$$

where the *E*_*loc*_*(ω)* and *E*_*inc*_*(ω)* are the E and E_0_ in the FDTD calculations, respectively. Therefore, the EM enhancement of the total EF was 1.08 × 10^6^, which was smaller than the value obtained from the experiment (2.8 × 10^6^). It is probably caused for two reasons. Initially, the nanopillars were more regular and the Au nanoislands were spherical with a smooth surface, which did not correspond with the complexity of the actual substrate microstructure. In addition, the CM mechanism may also contribute to the EF through the dynamic charge transfer effect between the nanopillars and R6G. The 3D-FDTD simulation results reveal that the SERS enhancement is actually due to the EM resonant excitation of LSPR, whereas the CM mechanism is necessary for the intensive study between the nanoscale structures and adsorbed molecules on the interface.

### Stability and reproducibility of the SERS substrate

**Figure 5:**
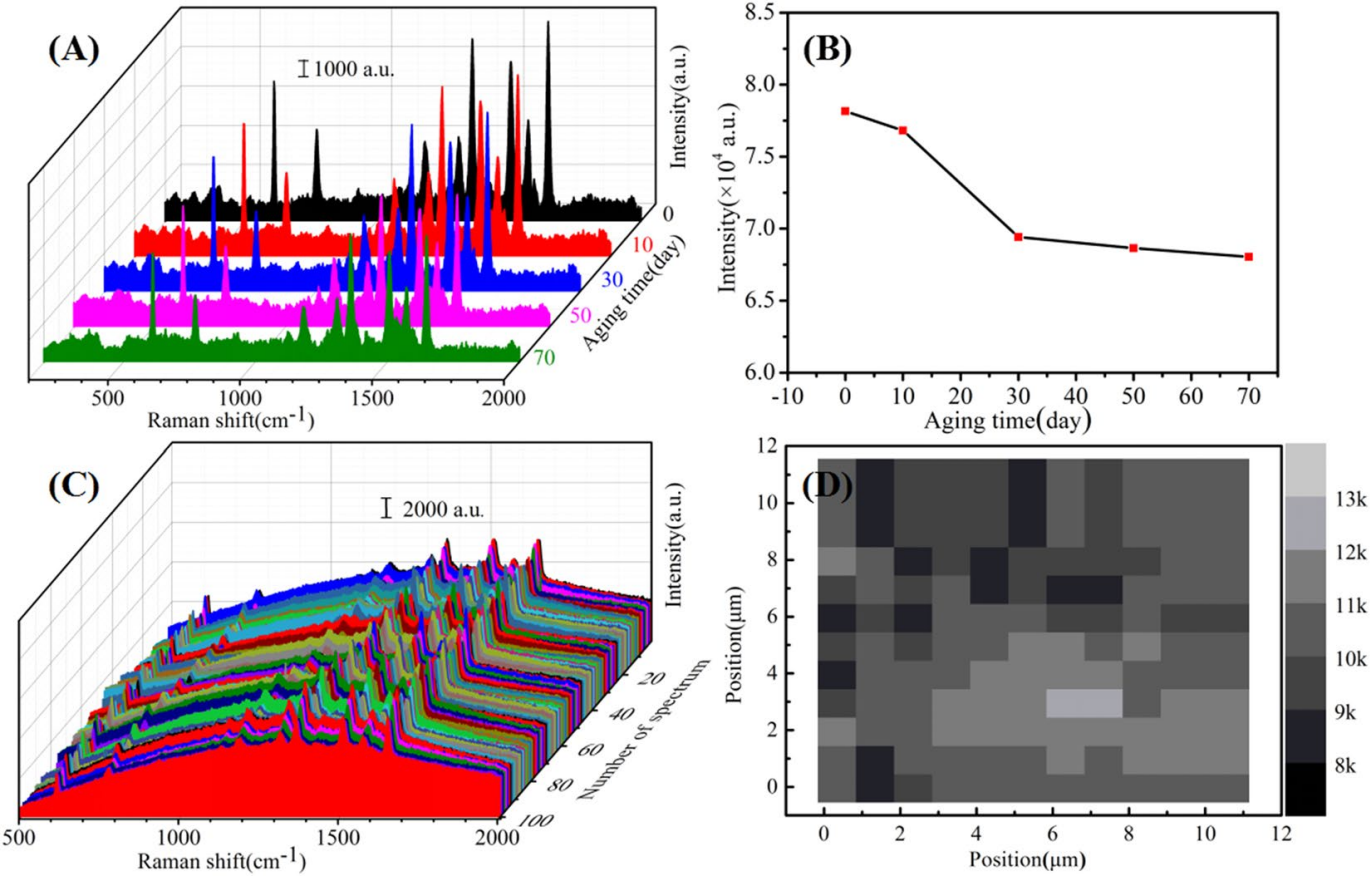
(**A**) SERS spectra of 10^−3^ M detected on Au/DW-45 substrate with different periods; (**B**) Plot of Raman intensities of R6G at 610 cm^−1^ versus different detection time; (**C**) SERS spectra of 10^−6^ M R6G obtained from 100 randomly selected spots on Au/DW-45 substrate; (**D**) the Raman mapping viewer (step size is 1 μm, 10 × 10 = 100 μm^2^) after the target molecule of R6G dried on the Au/DW-45, here the brightness is proportional to the signal integral intensity centered at 1361 cm^−1^.

In practical application, stability is an important parameter in high-performance substrates mainly because stable substrates can effectively reduce the waste of noble metals. In order to evaluate the stability of the Au/DW-45 substrates, the time-dependent SERS measurements were performed at room temperature. Figure 5(A) shows the 70 days stability tests of the Au/DW-45 substrates under the same experimental conditions. Obviously, the signal intensities of the 10^−3^ M R6G fell with varying degrees in different aging time. Fig. 5(B) exhibits the decay of the SERS intensities of R6G at 610 cm^−1^ as a function of aging time. In the first ten days, the signal intensity of the R6G decreased only by 1.71%. After aging for 70 days, the signal intensities trended to be stable and the SERS intensity fell by 12.9% compared to the intensity of fresh Au/DW-45 substrate. This remarkable stability reveals that compared to Ag-based SERS substrates^41,42^, gold is an ideal material in SERS application for its resistance to oxidation and this result further indicates that the Au nanoisland and DW are steadily combined without abscission by DC magnetron sputtering technique.

Apart from sensitivity and stability, good reproducibility of Raman signals is another indispensable requirement in practical applications of SERS technique. SERS spectra of R6G molecules with the concentration of 10^−6^ M from 100 different spots were shown in Fig. 5(C). Obviously, the Raman spectra had neither a significant shift of characteristic peaks nor changes of Raman intensities. In this case, the relative standard deviation (RSD) values of the intensities at the major peaks of R6G were shown in Table 2, indicating a good reproducibility across the entire area of the optimized Au/DW-45 substrates^43^. Meanwhile, the variation of intensity was within 8.3%, which was lower than those in previous work^44,45^. The result further demonstrates that DC magnetron sputtering technique owns the advantage in preparing high-performance SERS substrates. To further confirm the point-to-point reproducibility of Au/DW-45 substrates, a 10 μm × 10 μm = 100 μm^2^ area with a step size of 1 μm was randomly selected during SERS measurement and the point-to-point Raman mapping was presented in Fig. 5(D). It should be noted that the luminance of the grid was proportional to the signal intensity at 1361 cm^−1^ for 10^−6^ M R6G. These results clearly demonstrate that the as-prepared Au/DW-45 hybrids exhibited high uniformity and excellent reproducibility over the entire surface area as high-performance SERS substrates.

**Table 2:** RSD values at different major peaks of R6G.

Raman peaks (cm^−1^)	610	774	1187	1310	1361	1510	1572	1647
RSD values	7.8%	8.1%	8.3%	7.42%	7.73%	7.13%	5.75%	6.22%

### Detection of pesticide residues collected from tomato peels

**Figure 6:**
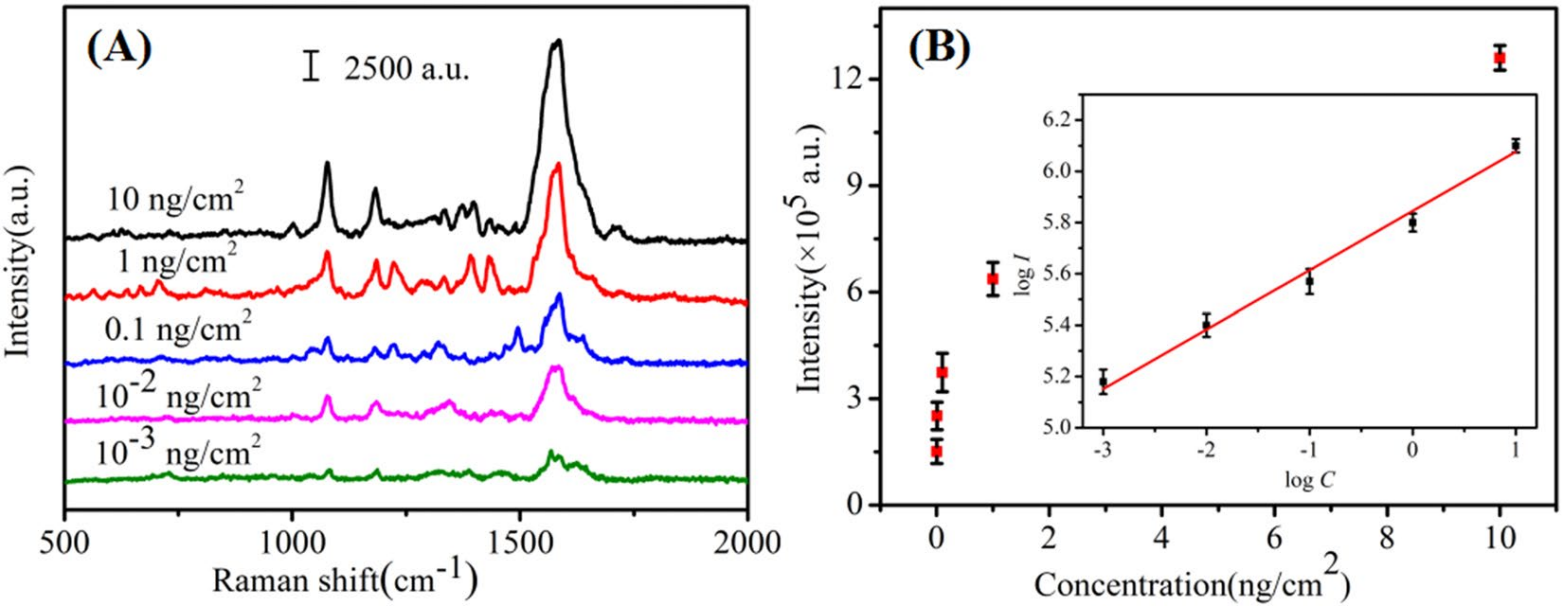
(**A**) SERS spectra of cypermethrin with concentrations of 10 ng/cm^2^–10^−3^ ng/cm^2^ collected from the surface of tomato peels; (**B**) Raman intensity of cypermethrin from tomato peels samples at 1584 cm^−1^ by using Au/DW-45 substrate and the inset is the quantitative logarithmic relation curve.

Pesticide is mainly used to prevent the agricultural production from diseases and insect pests and regulate the growth of crops. The use of pesticides can help to increase yields and increase farmers’ income, but the problem of pesticide residues cann’t be ignored. If the pesticide residues exceed the standard, it will pose a serious threat to the health of the human beings. In recent years, food safety has become a social focus problem, and pesticide residue detection is one of the important guarantee technologies for food safety^46^. Cypermethrin, as a kind of pesticide, has been wide used as an insecticide on the field of tea, fruit and vegetables. When the intake amount of cypermethrin is large, it can cause headache, dizziness, nausea, vomiting, trembling hands, convulsions, coma and even shock^47^. Therefore, seeking a facile approach to sensitively detecting cypermethrin is necessary. As we analyzed above, Au/DW-45 substrate showed high sensitivity, stability and reproducibility which satisfied the demands of solving the problem of pesticide residue in the real world. What’s more, the Au/DW-45 substrates are more flexible than other traditional rigid SERS substrates because they can be made into arbitrary curved surfaces or cut into specific sizes which require for nonplanar or curving surfaces. Therefore, the Au/DW-45 SERS platform was adopted to directly detect the trace of cypermethrin on tomato peels via a simple “press and peel off” method. First, the tomatoes were rinsed with deionized water and ethanol for three times to remove the pollutants from the surface of tomato peels. Second, the cleaned tomatoes were peeled by a fruit knife and cut into nearly uniform squares of 1 × 1 cm^2^. Afterward, 10 μL of the prepared cypermethrin solutions with different concentrations (10 ng/cm^2^–10^−3^ ng/cm^2^) were directly sprayed onto the peels. After natural evaporation at room temperature, a drop (10 μL) of ethanol solution was deposited onto each pre-treated sample. In this step, ethanol played a role in extracting pesticide residues. Finally, the SERS substrate was pressed to the samples until completely dry and then peeled off for Raman analysis. The variation of the Raman signal of cypermethrin with different concentrations was shown in Fig. 6(A). The signals observed at 1076, 1182, 1397 and 1586 cm^−1^ were the fingerprint peaks of cypermethrin. The Raman spectra of 10^−3^ ng/cm^2^ cypermethrin shows that the main peaks (1076, 1182, 1586 cm^−1^) of cypermethrin can still be distinguished at this ultra low concentration. It is indicated that the LOD of cypermethrin was estimated to be around 10^−3^ ng/cm^2^ for the Au/DW-45 substrates. Based on the experimental data of 10 independent measurements, the tight relationship between the SERS integrated intensities of the peaks centered at 1584 cm^−1^ and the concentrations was plotted in Fig. 6(B). When the concentrations and the intensities were all transformed into a logarithm scale, the response was almost linear (R^2^ = 0.987) over the concentration range as shown in the inset in Fig. 6(B). In a word, this kind of high-performance SERS substrate can be applied to rapidly detect other label-free organic molecules in real samples.

## Conclusion

In summary, Au nanoislands were successfully grown on the surface of DW by employing the DC magnetron sputtering system. By controlling the critical parameters of sputtering time, the fabricated 3D biomimetic arrays were optimized in order to yield substrates developing both SERS performance and practical application. 45 min-Au decorated DW SERS substrates were able to detect R6G molecular probes down to 1 × 10^−7^ M with good reproducibility (RSD less than 8.3%). The experimental results of time-stability showed that the Au/DW-45 substrate can still obtain good SERS signals after 70 days’ aging in room temperature, conforming that the Au/DW-45 substrate is stable. Furthermore, rapid SERS detection was investigated when the flexible Au/DW-45 substrate was used in collecting cypermethrin from tomato peels based on “press and peel off” approach with the LOD as low as 10^−3^ ng/cm^2^. In a word, the presented SERS substrate has advantages related to flexibility, good stability, reproducibility and rapid detection, which is expected to find potential applications in food safety.
